# Adding mechanical vibration to a half squat with different ballasts and rhythms increases movement variability

**DOI:** 10.1371/journal.pone.0284863

**Published:** 2023-07-27

**Authors:** Sílvia Tuyà Viñas, Bruno Fernández-Valdés Villa, Carla Pérez-Chirinos Buxadé, Mónica Morral-Yepes, Lucas del Campo Montoliu, Gerard Moras Feliu

**Affiliations:** 1 Department of Sports Performance, Institut Nacional d’Educació Física de Catalunya (INEFC), Universitat de Barcelona (UB), Barcelona, Spain; 2 Department of Health Sciences, Research Group in Technology Applied to High Performance and Health, TecnoCampus, Universitat Pompeu Fabra, Barcelona, Spain; 3 Department of Strength and Conditioning, Futbol Club Barcelona, Sant Joan Despí, Spain; Università degli Studi di Milano: Universita degli Studi di Milano, ITALY

## Abstract

The aim of this study was to determine whether whole body vibration increases movement variability while performing a half squat with different ballasts and rhythms through entropy. A total of 12 male athletes (age: 21.24 ± 2.35 years, height: 176.83 ± 5.80 cm, body mass: 70.63 ± 8.58 kg) performed a half squat with weighted vest, dumbbells and bar with weights suspended with elastic bands, with and without vibration at the squat rhythm of 40 and 60 bpm. Each ballast corresponded to 15% of the body mass. The movement variability was analysed by calculating the sample entropy of the acceleration signal, recorded at the waist using an accelerometer. With vibration, differences were found between weighted vest and dumbbells (t(121) = -8.81, p < 0.001 at 40 bpm; t(121) = -8.18, p < 0.001 at 60 bpm) and between weighted vest and bar at both rhythms (t(121) = -8.96, p < 0.001 at 40 bpm; t(121) = -8.83, p < 0.001 at 60 bpm). Furthermore, a higher sample entropy was obtained at 40 bpm with all ballasts (t(121) = 5.65, p < 0.001 with weighted vest; t(121) = 6.27, p < 0.001 with dumbbells; t(121) = 5.78, p < 0.001 with bar). No differences were found without vibration. These findings reveal that adding mechanical vibration to a half squat produces a non-proportional increase in movement variability, being larger when the ballast is placed on the upper limbs and when performed at a slow rhythm.

## Introduction

Over the last years, the application of instability in strength training to produce a destabilising effect on the body has become very popular. Different devices have been used for this purpose to increase muscular demands and to enhance neuromuscular adaptations, for example free weights [1] or unstable surfaces [2, 3]. Recently, a new way of using free weights to increase the destabilising effect on the body during exercise has been introduced. This involves applying external loading to a bar suspending it by elastic bands [4]. In addition, some research has studied mechanical vibration and its effect on balance. Sierra-Guzmán et al. [5] obtained balance improvements in one of the tests after a training programme with whole body vibration (WBV) on an unstable surface. Other authors found an improvement in gait initiation balance after acute application of WBV [6]. These results suggest that mechanical vibration might be a stimulus capable of causing postural destabilisation.

Adding mechanical vibration in exercises is a way to complement traditional strength training for athletes, healthy people, elderly people, and health-compromised individuals [5, 7]. The most popular vibration modality applied to lower limbs exercise is WBV [7]. One of its main properties is the improvement of specific aspects of neuromuscular performance such as strength [8], stability [5, 6] or muscle activity as evaluated with surface electromyography (EMG) [9]. Regarding jumping, agility and sprint performance, the data obtained from the meta-analysis by Minhaj et al. [10], do not support the use of WBV in isolation. Furthermore, WBV exercises can be combined with different training devices such as ballasts [11], suspension device straps [2] and unstable surfaces [5].

Movement variability (MV) is an indicator of the motor control that assesses movement regularity [12]. It is inherent in motor system and is therefore present in any motor action. However, when the athlete faces new or complex situations, the central nervous system is forced to find an optimised motor solution, exploring the dimensionality provided by the multitude of degrees of freedom, which causes an increase in its MV [13]. In particular, this increase in MV is what enables motor adaptation to changes in the environment, thus it is considered a tool to stimulate learning [14]. In that regard, the application of an instability stimulus in strength training that produces a destabilising effect on the body tends to increase MV [15]. When an athlete masters an exercise the MV tends to decrease, and its training potential is reduced. In this case, to ensure that exercise remains useful in promoting improvements in the athlete, it would be desirable to introduce constraints that induce an increase in MV [16]. These constraints applied to the exercises become important in sports, especially in team and situation sports, characterised by unpredictable and changing environments [17]. These athletes must continuously perceive and interpret external and internal information to adjust their actions and obtain effective and efficient solutions [18]. This results in variable and unrepeatable actions of different intensity and directionality [19]. Therefore, strength training should not focus on improving only conditional performance, which is mainly focused on quantitative assessments [20].

To date, the measurement of the effects of destabilising devices has been addressed through different parameters as EMG activity [1, 21, 22], external load moved in different exercises [4], movement time and amplitude [23], movement speed [24, 25], displacement of the centre of pressures and acceleration of the centre of mass measured trough a force plate [6], kinetic and kinematic variables captured from 3D movement analysis [26] or trunk acceleration [15, 16, 27–29]. Regarding trunk acceleration, several authors used accelerometers placed in the lower back to measure different tasks [27, 28] and found high correlation with balance measures obtained through force plates [30]. Most analyses have been done using measurements for linear systems, which quantify the magnitude of variations in a time series. However, to obtain information about body MV, measurements for non-linear systems must be used, as they quantify the regularity in a time series [31]. From this perspective, different tools have been used, one of them is the sample entropy (SampEn) [15, 16, 29]. Therefore, the aim of this study was to determine whether WBV increases MV while performing a half squat with different ballasts and rhythms through entropy. It was hypothesized that (a) adding mechanical vibration to a half squat would produce an increase of MV, (b) the bar with weights suspended with elastic bands would be the ballast that produced the highest MV and (c) the rhythm that would produce the highest MV would be 60 bpm.

## Materials and methods

### Study design and participants

**Fig 1:**
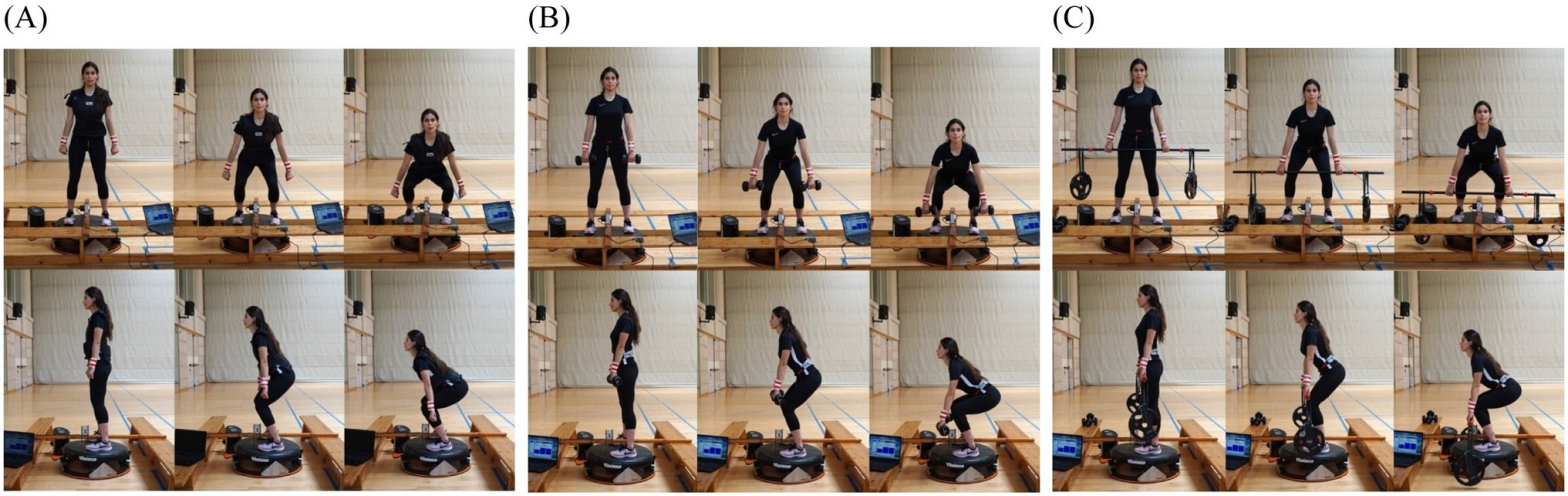
Scenario of the squat with the three different ballasts, (A) weighted vest, (B) dumbbells and (C) bar with weights suspended by elastic bands. The structure shown in the image was designed to fix the encoder and to prevent its contact with the vibration platform. Images with permission of the executor.

The current study used a single-group within-subject factorial experimental design to determine the effect of different constraints applied to a half squat. In total, 12 male amateur athletes in different sports voluntarily participated in the study ((mean ± SD) age: 21.24 ± 2.35 years, height: 176.83 ± 5.80 cm, body mass: 70.63 ± 8.58 kg, strength-training frequency: 2.50 ± 1.93 days/week). All athletes were informed of the benefits and risks of the investigation prior to signing the informed consent. The model in the images in Fig 1 gave informed written consent to publish her images. The procedures complied with the Declaration of Helsinki (2013) and were approved by the local ethics committee (036/CEICGC/2021).

### Data collection

All the athletes attended a total of three sessions, conducted at the same time of day. To minimize the influence of fatigue, athletes were asked to abstain from exercise for 48 hours before testing. Moreover, they were required to wear sportive poorly cushioned shoes, in order to minimize differences in vibration transmissibility between subjects.

The first session consisted of familiarization. After activation, participants performed one set of twelve repetitions of all half squat combinations upon a vibration platform (Vibalance 2.0, Byomedic System SLU, Spain) and practiced squat depth. To ensure proper squat depth, a thin elastic band was placed as a reference. This was adjusted to the height of the back of the thigh for each subject coinciding with the 90º of knee flexion. A goniometer was used for this purpose.

**Fig 2:**
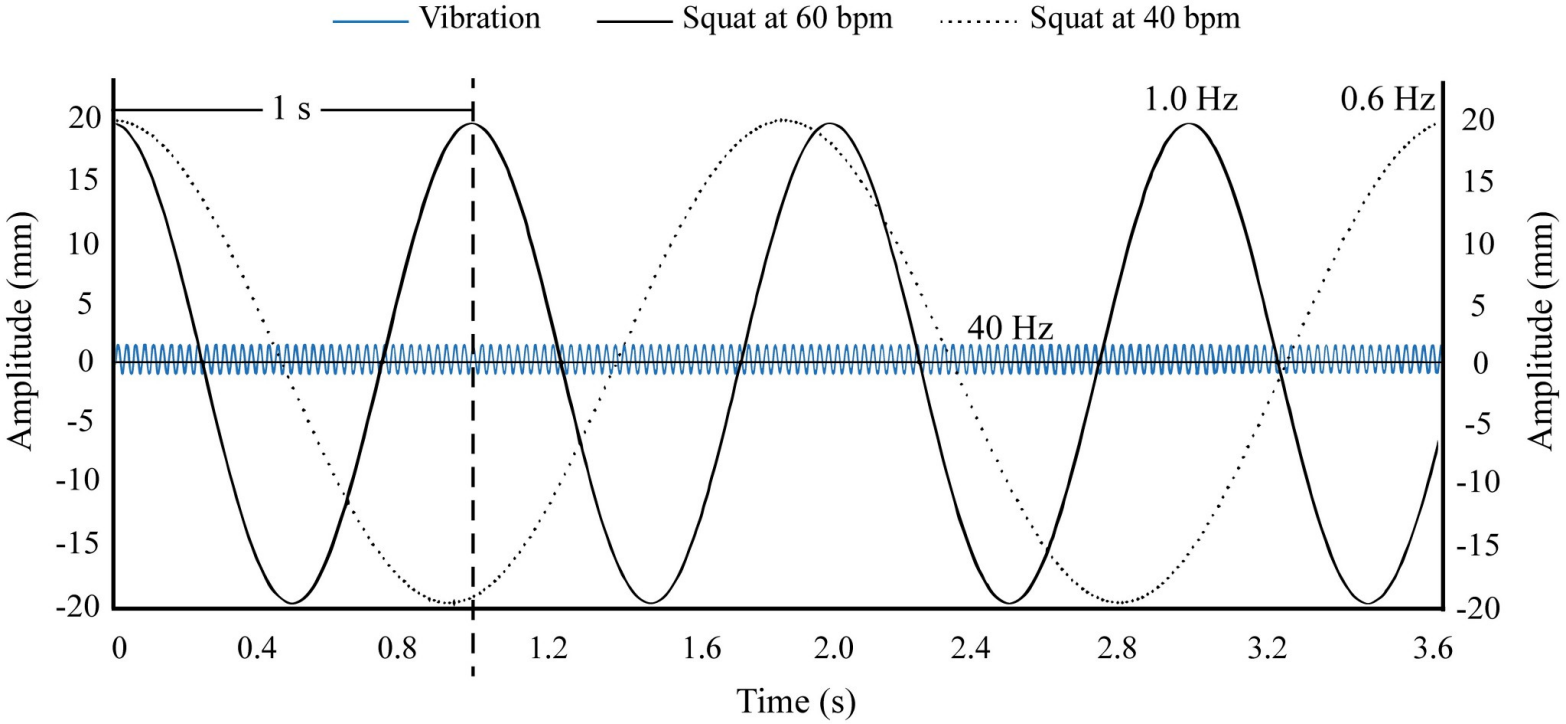
Diagram of the oscillations of mechanical vibration and vertical squat movement. It shows the amplitude (1.8 mm) and frequency of vibration (40 Hz) and the vertical movement of the subjects when performing the squat at both rhythms (40 and 60 bpm, corresponding to 0.6 and 1.0 Hz, respectively).

The following two sessions consisted of data collection. First, participants performed a 5 min activation protocol, which consisted of 3 min cycling followed by two sets of fifteen repetitions of bodyweight half squat. Thereafter, an inertial measurement units (IMU) device with an accelerometer (WIMU, Realtrack Systems, Almeria, Spain: weight: 70 g, size: 81 mm x 45 mm x 16 mm) was placed to all the athletes using an adjustable sports lycra belt, which fixed the device at the back of the waist, at L4-5 level. This position provided the best information about the movement of the whole body, as the location is close to the centre of mass [15, 27]. The IMU was set to a sampling frequency of 1000 Hz, and it was calibrated on a flat and even surface with the z-axis perpendicular to the surface. Each subject performed twelve sets of twelve repetitions of a half squat. Specifically, four sets were performed with each of the three different ballasts: weighted vest (WV), dumbbells (D) and bar with the weights suspended by elastic bands (B) (Fig 1). Each ballast corresponded to 15% of the body mass. Two of the four sets were performed without vibration and the other two with vibration, one of them at the squat rhythm of 40 bpm and the other at the squat rhythm of 60 bpm (0,6 and 1,0 Hz respectively) (Fig 2). The rhythm was controlled by a metronome [15], synchronizing the beep with the end of the concentric phase. The frequency of the vibration platform was set at 40 Hz and the amplitude was 1.8 mm. All sets were performed in random order with a minimum of 3 min rest in between. To perform the half squat, the arms remained extended under the body and slightly forward in order not to modify the acceleration movement pattern, and the knees had to reach a 90° flexion at the lowest point of the squat. A linear encoder (Chronojump Boscosystem^®^, Barcelona, Spain) placed between subject’s feet was used to control the depth of the squat, measuring the vertical displacement (Fig 1). All sets were recorded with a video camera (GoPro Hero 7).

Six central repetitions were selected after a visual check of the vertical displacement obtained with the encoder using the Chronojump software (version 1.8.0, Chronojump-Boscosystem, Spain) to verify the uniformity of the signal. Total acceleration was selected from the data obtained by the accelerometer, that is the modulus of the vector resulting from the sum of vectors of the acceleration of each axis (x, y, z). SPRO 951 software (version 1.0.0, Realtrack Systems, Spain) was used for the total acceleration signal treatment, that was synchronized with the video. The acceleration data was not filtered to accurately analyse the variability within the time series, as Craig et al. [32] did, following the recommendation of Mees and Judd [33]. The SampEn of each set was calculated according to Moras et al. [15] and following procedures established by Matlab^®^ (version R2020a, The MathWorks, Massachusetts, USA).

### Statistical analyses

Data were not normally distributed (Shapiro-Wilk test was performed). In order to address this, SampEn was log-transformed. Subsequently, a linear mixed model was performed on the log-transformed dependent variable SampEn with ballasts (WV, D and B), rhythms (40 and 60 bpm) and vibration conditions (with vibration and without vibration) and all interactions between factors as fixed effects and subject as the random effect. The degrees of freedom were corrected using the Kenward-Roger method. Finally, post-hoc test with Tukey adjustment for multiple comparisons was performed within factors. Also, comparisons were assessed via effect size (ES) test using 95% confidence intervals. Thresholds for ES were 0.2 trivial; 0.6 small; 1.2 moderate; 2.0 large; and > 2.0 very large. For all statistical tests, a nominal significance level of 5% (p-value < 0.05) was applied. The statistical analysis was performed using R (v4.1.2, R Foundation for Statistical Computing, Vienna, Austria).

## Results

The three main factors of the model (vibration condition, ballast and rhythm) were found to be statistically significant (F(1,121) = 1716.86, p < 0.001; F(2,121) = 27.28, p < 0.001; F(1,121) = 61.65, p < 0.001, respectively). In addition, the interactions between the vibration condition factor and the ballast factor, and between the vibration condition factor and the rhythm factor were significant (F(2,121) = 25.38, p < 0.001; F(1,121) = 43.58, p < 0.001, respectively), implying that the differences between the ballasts and between the rhythms depend on the condition of the vibration factor.

**Fig 3:**
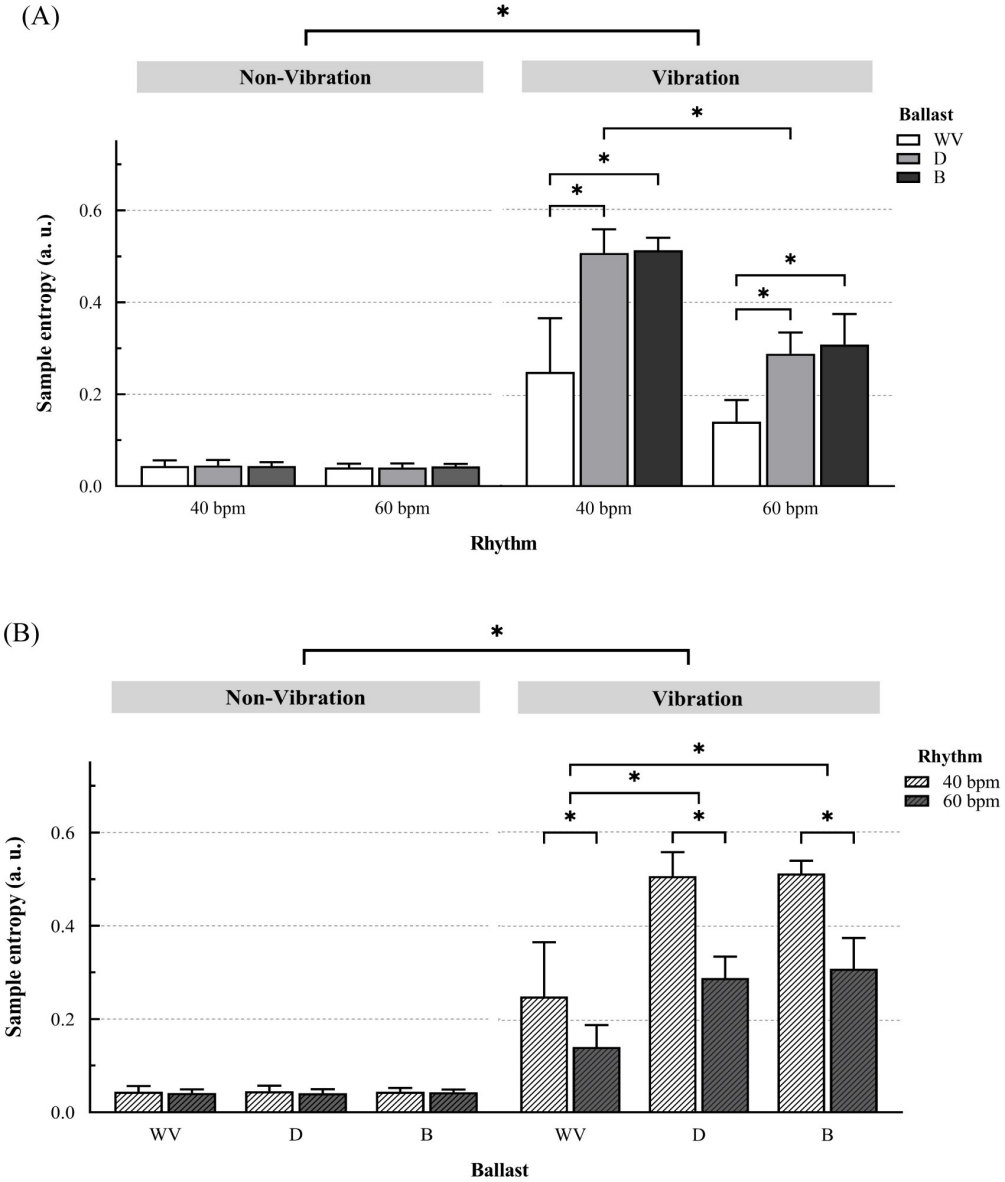
Comparison of sample entropy under the different conditions. It shows Sample entropy values (mean and 95% CI) of the squat with the three ballasts (WV, D and B) with (vibration) and without vibration (non-vibration) at both rhythms (40 and 60 bpm). Significance values come from the adjusted linear mixed model and only statistically significant differences are shown (p < 0.001), represented by *. SampEn: sample entropy, CI: confidence interval, WV: weighted vest, D: dumbbells, B: bar with weights suspended by elastic bands.

When comparing SampEn between the vibration conditions (with vibration vs without vibration, Fig 3), with vibration a higher entropy was shown with all ballasts with a very large ES (t(121) = 22.00, p < 0.001, ES = 4.82 for ballast WV; t(121) = 33.94, p < 0.001, ES = 7.44 for ballast D; t(121) = 34.13, p < 0.001, ES = 7.48 for ballast B). Analogously, a higher entropy was found in both rhythms when vibration was applied to the squat (t(121) = 41.44, p < 0.001, ES = 7.41 for rhythm of 40 bpm; t(121) = 32.10, p < 0.001, ES = 5.74 for rhythm of 60 bpm).

When comparing the ballasts (Fig 3A), it seems that differences were only found when vibration was applied. Differences were found at both rhythms and with a very large ES between WV and D (t(121) = -8.81, p < 0.001; ES: -2.73; IC95%: -3.43 –-2.03 at 40 bpm; t(121) = -8.18, p < 0.001; ES: -2.53; IC95%: -3.22 –-1.85 at 60 bpm) and between WV and B (t(121) = -8.96, p < 0.001; ES: -2.78; IC95%: -3.48 –-2.08 at 40 bpm; t(121) = -8.83, p < 0.001; ES: -2.74; IC95%: -3.44 –-2.04 at 60 bpm). No differences were observed between D and B in any of the rhythms. While without vibration there were no significant differences among ballasts.

**Fig 4:**
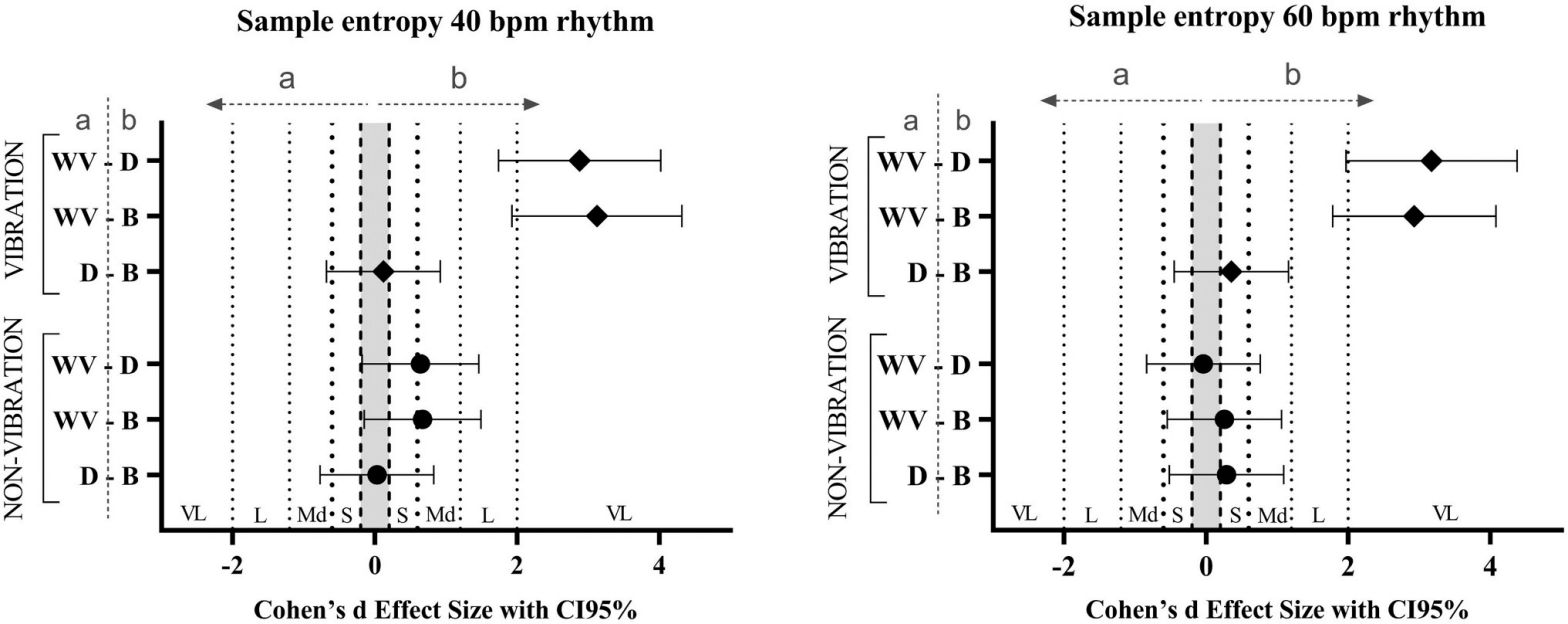
Effect size using Cohen’s *d* of the sample entropy differences between the ballasts. The error bars indicate the uncertainty in the changes of the average with a 95%CI. SampEn: sample entropy, CI: confidence interval, WV: weighted vest, D: dumbbells, B: bar with weights suspended by elastic bands, S: small, Md: moderate, L: large, VL: very large.

Regarding the rhythms (Fig 3B), the 40 bpm rhythm achieved a higher SampEn with all ballasts with vibration compared to the rhythm of 60 bpm, and the differences were large (t(121) = 5.65, p < 0.001; ES: 1.75; IC95%: 1.10–2.40 with WV; t(121) = 6.27, p < 0.001; ES: 1.94; IC95%: 1.29–2.60 with D; t(121) = 5.78, p < 0.001; ES: 1.79; IC95%: 1.14–2.44 with B) (Fig 4), whereas without vibration there were no significant differences.

## Discussion

The main finding of this study is that adding mechanical vibration to a half squat produces an increase in MV that is not proportional between the ballasts and between the rhythms analysed. According to the results of the present study, WBV by itself can increase movement irregularity of the athletes’ centre of mass, whereas this does not seem to occur when only the rhythm or the type of ballast is modified. However, it seems that mechanical vibration can interact with the effect of these other elements when combined, achieving different levels of destabilisation. The increase in MV during exercise with WBV should be considered as a facilitating feature of the adaptive capacity to the environment, which is necessary to improve the athlete’s performance [13]. Therefore, the inclusion of WBV in strength training seems to be a good option to achieve adaptations at different levels of the neuromuscular system, approaching the reality of the athletes [20]. To date it is known that muscle activity increases with the addition of mechanical vibration to static and dynamic exercises of upper and lower body [34]. Moreover, Arora et al. [35] found a large effect on muscle activity after 6 weeks of loaded back squat with WBV.

Experimental evidence supports the tonic vibration reflex as a mechanism underlying the neuromuscular response to WBV. In this regard, mechanical vibration has been shown to perturb postural regulation, via integrative mechanisms involving supraspinal structures. This results in increased phasic muscle activation [21]. Therefore, the increase in MV caused by mechanical vibration can be explained by the acute neuronal modulation that the vibratory stimulus produces in the central nervous system. Specifically, a change in motor command has been found to increase excitability at the supraspinal level, analogous to inhibition at the spinal level. Thus, there is an increase in cortical activity to control body position [36]. This behaviour implies an alteration in the configuration of postural responses in relation to external perturbations [37]. In any case, we can state that the MV obtained was the product of the vibratory stimulus produced by the vibration platform and the modulation of the central nervous system.

Regarding the ballast, an interaction between the vibration condition and the ballast factors was found. Differences were only found with vibration between WV and D, and WV and B. Therefore, WBV forced postural destabilisation, especially with D and B. This behaviour could be explained, partly, due to the aerial phase that a rigid body undergoes when placed on top of a synchronous vibration platform [7]. This effect was already suggested by Sierra-Guzmán et al. [5] who investigated it through a balance analysis. During the exposure to mechanical vibration, the body separates and collides again with the platform, causing a non-contact phase that decreases grip and increases the difficulty of control that can lead to slippage, especially when the base of support is smaller. In addition, these aerial phases can lead to the omission of one or several vibration cycles and, consequently, to the generation of subharmonic frequencies in the body [7]. Furthermore, from a physics point of view, the human body in a standing position can be compared to an inverted pendulum. When it receives a perturbation on the vertical axis it deviates from the equilibrium point [38]. The multiple body segments act as inverted pendulums themselves, transferring the perturbations to the other segments during movement. Translating this concept into the reality of this study, when using the WV ballast, the load was closer to the centre of mass having a lower effect on the inverted pendulum than if the load is held in the hands. Therefore, it is logical that the MV was higher with D and B than with WV, making it more difficult to perform the movement with motor control. Surprisingly, no differences were found between D and B with vibration, although it would appear that B would be the most destabilising ballast. In this respect, Kohler et al. [3] and Saeterbakken et al. [1] defined the conventional bar as a more stable ballast than D in an overhead shoulder press and a chest press, respectively. Lawrence et al. [4] also determined the conventional bar as more stable than B in a bench press. Therefore, previous research, indicates that D and B can generate destabilisation, although no literature has been found comparing them to each other. It should be noted that all these studies were performed without mechanical vibration. Thus, it seems that in ballast B of the present study, the bar may have reduced the destabilising effect of the elastic bands in a way that it ultimately generates the same effect as D. Probably, if instead of using a bar, weights had been taken directly by the hands through the elastic bands, a higher MV would have been obtained than with the ballast D.

In contrast to similar studies without vibration [24, 25], the present study reported that the type of ballast used did not produce differences in the MV performing the squat without vibration at both rhythms. This means that the ability to control the centre of mass had to be similar in the squat performed with the three ballasts. Thus, the ballast and load used in the present study were not enough destabilising to produce differences in MV without vibration. Future research should investigate whether differences appear when the exercise is performed at higher loads. In the same line of this research, but analysing the EMG activity, Wu et al. [22] also found no differences when comparing a squat with WV and D. Other studies that reported changes in EMG activation or in velocity, used as destabilising devices an Attitube (a cylinder half-filled with water) [24] and the same bar as B, used in the present study, although in this case the participants carried it on their shoulders during a back squat instead of holding it in their hands [25].

Regarding the rhythm, an interaction was shown between the vibration condition and the rhythm factors. Differences in MV were found between the two rhythms when vibration was applied to the squat with the three ballasts, being higher at the slower rhythm, of 40 bpm. This fact could be explained by the relationship between the rhythm of the athlete’s movement and the vibration frequency of the platform. Performing the squat at the rhythm of 60 bpm means that in one repetition the athlete is exposed to 40 vibration cycles and at the rhythm of 40 bpm the athlete is exposed to 66.7 vibration cycles. This implies that at the slowest rhythm the athlete is exposed to more vibration cycles than at the fastest rhythm. Accepting that each vibration cycle involves a vertical force that tends to separate the body from the platform causing a non-contact phase that decreases grip and increases the difficulty of control [7], it is not surprising that the athletes had more requirements to rebalance themselves at the slower rhythm.

On the contrary, in the absence of mechanical vibration, the MV was similar with the two rhythms used. In scientific literature, studies have analysed this from simpler movements than in the present study and the results are diverse. Salmond et al. [39], in a task involving the upper limbs, also found no differences between rhythms. Contrarily, Park et al. [40], demonstrated that slower oscillatory movements performed with the arm deviated more from harmonicity. This concept of harmonicity was defined as the proximity of the trajectory to the sinusoidal and calculated from the time and amplitude of the movement [23]. These results could be attributed to the fact that humans do not master moving slowly or do not feel comfortable. Other studies that analysed tasks involving moving a handheld dowel back and forth between two large targets in time with a metronome, demonstrated changes in dynamic primitive according to the interval of time. In longer time periods, participants avoided moving slowly and moved acceptably fast, making longer pauses at each target [41]. In the same way, Park et al. [40] reported that the subjects tended to achieve discrete submovements and that the trajectory was less smooth, whereas when the rhythm increases, movements cannot be kept discrete and become oscillatory [42]. These results could support the proposal that motor control is based on dynamic primitives [40, 43] and these are limited [23]. In addition, these two different dynamic primitives, rhythmic and discrete movements, are associated with distinct areas of the motor cortex [43]. In any case, this inability to perform continuous slow movements in a smooth and regular pattern reflects the human nature and its limitations at the motor control level [40] being magnified in more destabilising situations. Nevertheless, it seems that in the present study, without applying mechanical vibration to the athlete’s body, the 40 bpm rhythm was not slow enough to detect a loss of harmonicity or the achievement of discrete submovements that result in less regular movement.

Whilst this study showed important and insightful results on the effects of mechanical vibration combined with other elements on movement regularity, some limitations must be acknowledged. Firstly, the results were obtained when analysing a half squat, but it is not known whether they would be applicable to other strength exercises. Secondly, being aware that the frequency and amplitude of vibration influence aspects such as muscle activation, it is unknown whether they could also influence MV. In this research, a frequency of 40 Hz and an amplitude of 1.8 mm were chosen as being within the range described by other authors as producing more electromyographic activation. Finally, a low external resistance was used that did not cause a high level of metabolic fatigue. In this sense, it is possible that higher external resistances could reduce the air phase produced by the vibration and thus influence the regularity of the acceleration signal. In relation to this, it would be interesting for future research to analyse the MV when using other external resistances, frequencies and vibration amplitudes, and their effects after a training programme.

## Conclusions

In conclusion, the present study provides evidence that adding mechanical vibration to a half squat produces an increase in MV. Furthermore, this increase in MV is not proportional between the ballasts and between the rhythms analysed. The largest differences compared to the exercise without vibration were found in D and B ballasts and in the 40 bpm rhythm. These results show for the first time that exercise with WBV could have a destabilising effect, apart from the effects on muscle activation that many authors have already extensively investigated.

### Practical applications

When strength and conditioning coaches aim to increase MV, it is preferable to perform the exercises with mechanical vibration, applied through the feet by means of a vibration platform, and to use as ballast D or B, rather than WV. In turn, an increase in this MV will be achieved if slow performance rhythms are selected.

It is recommended to include destabilisation in strength exercises in order to increase the training potential of the exercises and to improve the adaptive capacity of the athletes. This will be particularly relevant in team and situational sports due to their variable nature.

It is preferable to introduce this type of exercise with high MV progressively and in combination with other more stable exercises, and always according to the level and characteristics of each athlete.

Entropy seems to be a suitable tool to analyse the destabilizing effect of constraints such as WBV, type of ballast and rhythm.

## Supporting information

S1 Data Raw data of total acceleration and sample entropy calculation for all squat sets of all subjects in the study. P: participant, ACLT: total acceleration, SampEn: sample entropy, NoVib: without vibration, Vib: with vibration, WV: weighted vest, D: dumbbells, B: bar with weights suspended by elastic bands.
(XLSX)
